# Supplementary material for: Gel-assisted mass spectrometry imaging enables sub-micrometer spatial lipidomics
Source: Nat Commun. 2024 Jun 12;15:5036. doi: 10.1038/s41467-024-49384-w (PMC11169460; doi:10.1038/s41467-024-49384-w)
Supplement: Supplementary file 1 — Supplementary Information [file 41467_2024_49384_MOESM1_ESM.pdf]

Supplementary Information for

Gel-assisted mass spectrometry imaging enables sub-micrometer spatial lipidomics

Yat Ho Chan *et al.*

Corresponding author: Ruixuan Gao, gaor@uic.edu

The PDF file includes:

Supplementary Figs. 1 to 14

Supplementary Figures

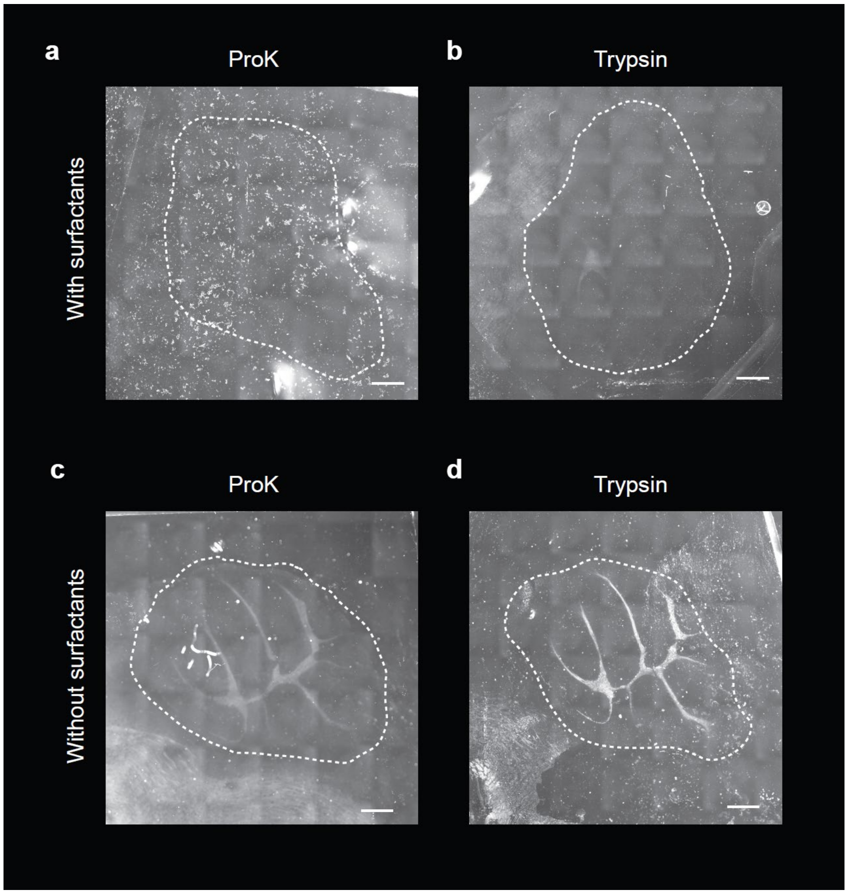

Supplementary Fig. 1: Surfactant treatment and lipid retention in thin tissue slices. Post-expansion images of ~25 µm thick mouse brain slices digested using a (**a**) proteinase K (proK, with surfactants), (**b**) trypsin (with surfactants), (**c**) proK (without surfactants), (**d**) trypsin (without surfactants) digestion buffer. The optical images were obtained using wide-field detection and oblique white-light LED illumination. The broken line indicates the location of the cerebellum. Scale bars, 500 µm (1.9 mm). Here and after, unless otherwise noted, scale bars are provided at the pre-expansion scale (with the corresponding post-expansion size indicated in the brackets).

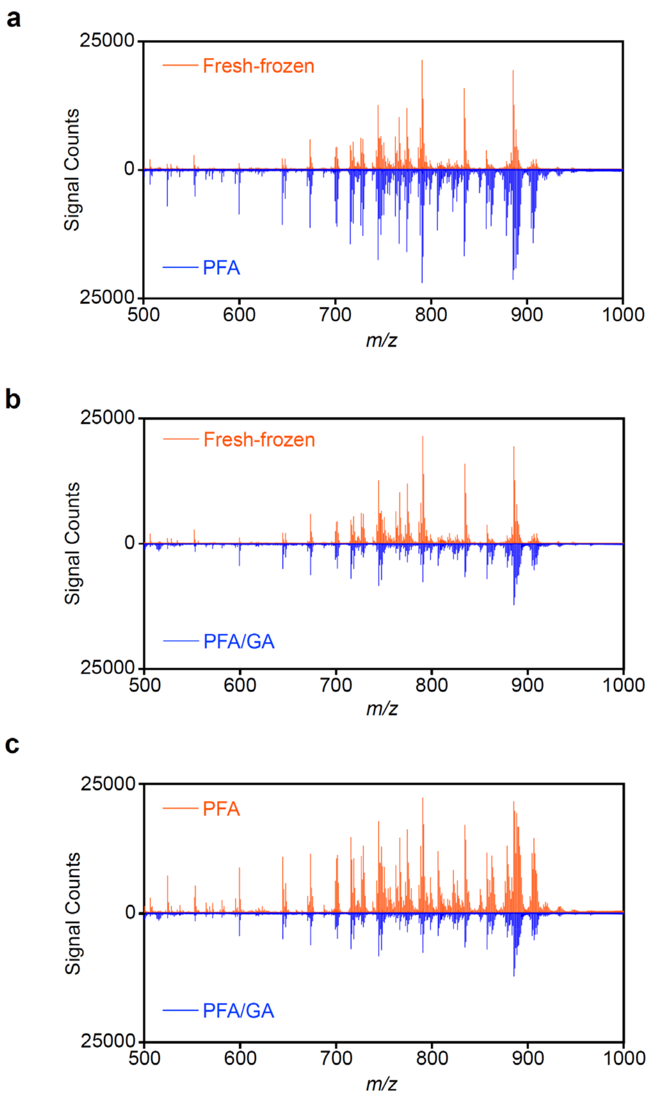

Supplementary Fig. 2: Averaged mass spectra (*m/z* 500-1000) of fresh-frozen, PFA-fixed (non-expanded), and PFA/GA-fixed (non-expanded) mouse cerebellum. Comparisons between (**a**) fresh-frozen and PFA-fixed, (**b**) fresh-frozen and PFA/GA-fixed, and (**c**) PFA and PFA/GA-fixed samples are shown. The mass spectra were collected using an Applied Biosystems SCIEX 4800 MALDI TOF/TOF Analyzer ("AB SCIEX 4800") with an instrument pixel size of 100 µm. Source data are provided as a Source Data file.

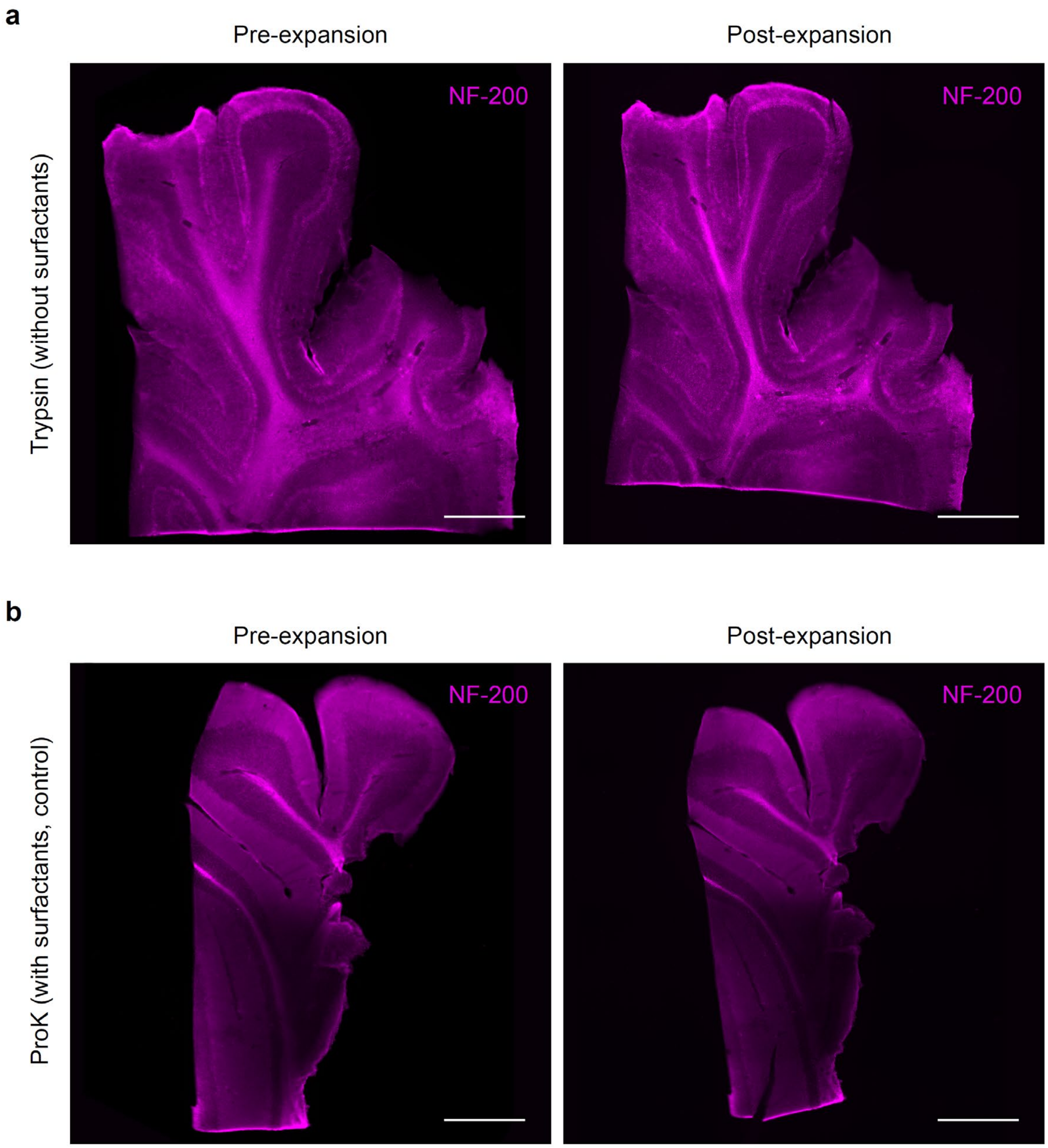

Supplementary Fig. 3: Trypsin and proteinase K (proK) digestion of thin tissue slices. Pre- (left) and post-(right) expansion images of ~40 µm thick mouse brain slices digested using a (**a**) trypsin (without surfactants) and (**b**) proK (with surfactants, as control) digestion buffer. The brain slices were fluorescently labeled using neurofilament (NF)-200 antibodies. Scale bars, 500 µm (1.9 mm).

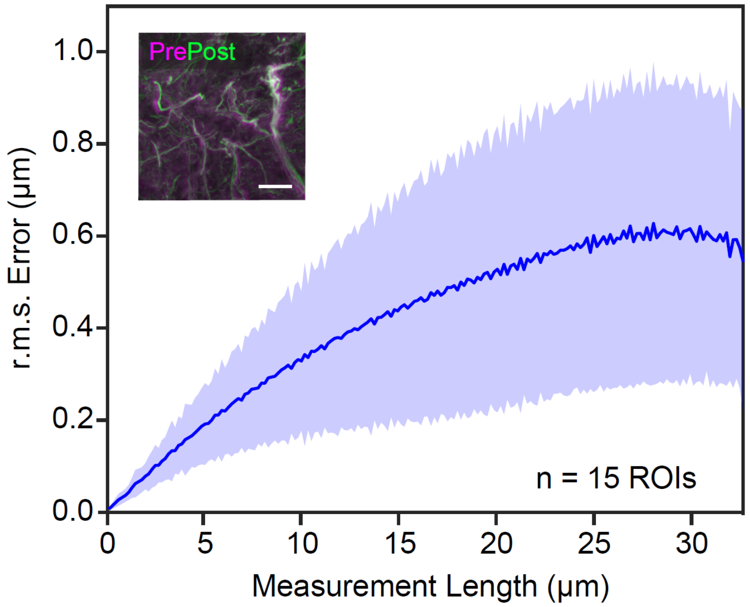

Supplementary Fig. 4: Expansion isotropy analysis. Root-mean-square (r.m.s.) expansion error of ~25 µm thick mouse brain slices homogenized by trypsin digestion (without surfactants) [blue line, mean; shaded area, standard deviation; n = 15 regions of interest (ROIs) from three brain slices from two animals]. Inset: Non-rigidly registered and overlaid pre- (magenta) and post-expansion (green) images used for the r.m.s. error analysis. Scale bar, 5 µm (15 µm). Source data are provided as a Source Data file.

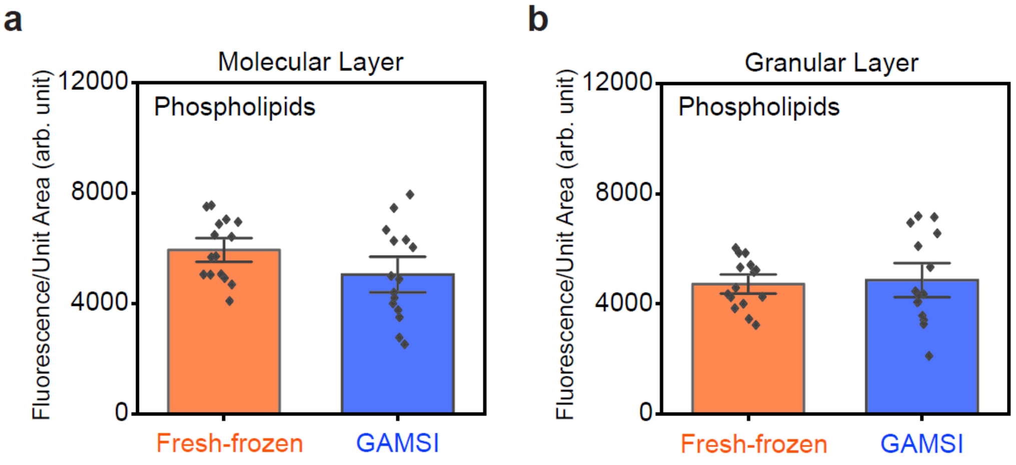

Supplementary Fig. 5: Lipid retention in GAMSI-processed mouse brain slices. Fluorescence intensity per normalized unit tissue area of fresh-frozen and GAMSI-processed (PFA/GA-fixed) mouse cerebellum (**a**) molecular layer and (**b**) granular layer, fluorescently labeled using a phospholipid dye [bar height, mean; black dots, individual data points; error bar, standard error of the mean (SEM); n = 15 regions of interest (ROIs) from three brain slices from one animal]. The unit tissue area was normalized to the pre-expansion scale. Source data are provided as a Source Data file.

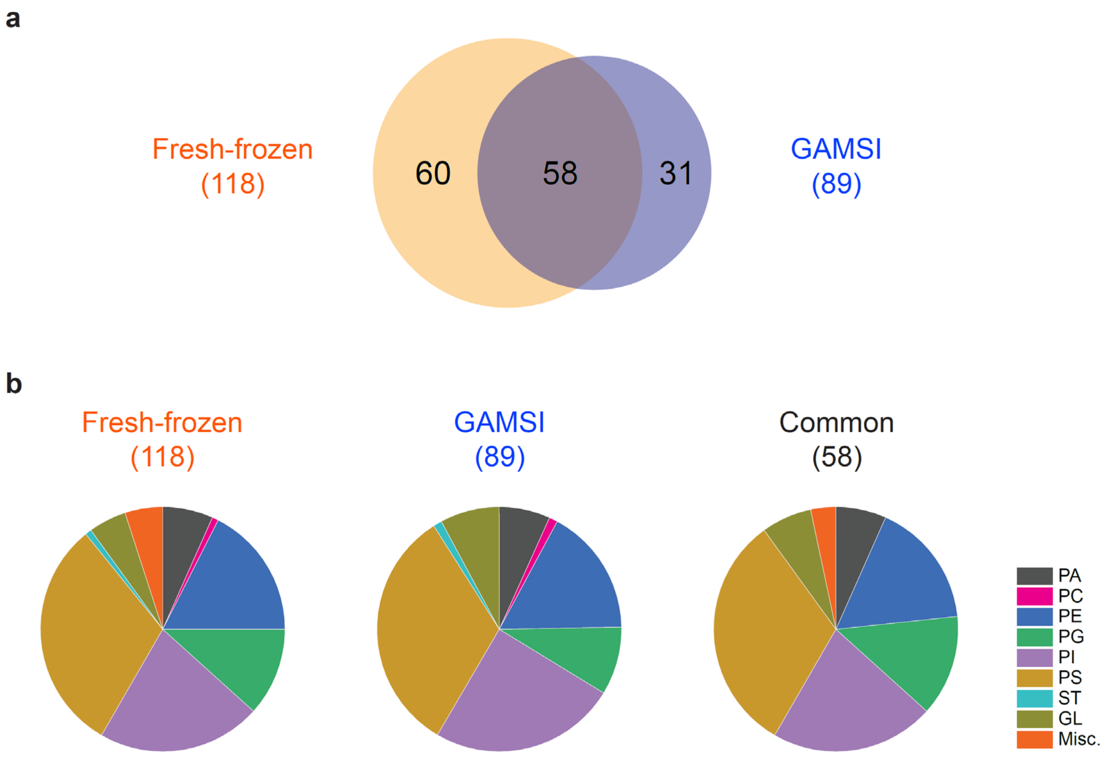

Supplementary Fig. 6: Lipid profile of fresh-frozen and GAMSI sample (AB SCIEX 4800). a, Venn diagram showing common and different lipid peaks from averaged mass spectra of fresh-frozen and GAMSI-processed (PFA-fixed) mouse cerebellum using an AB SCIEX 4800. All lipid assignments were made by comparing the lipid peaks to the LIPID MAPS database with an allowed mass tolerance of *m/z* = +/- 0.05. **b,** Pie chart showing the chemical composition of all the fresh-frozen peaks (left), GAMSI peaks (center), and common peaks (right). Instrument pixel size was set at 100 µm. PA: phosphatidic acid; PC: phosphatidylcholines; PE: phosphatidylethanolamine; PG: phosphatidylglycerol; PI: phosphatidylinositol; PS: phosphatidylserine; ST: sterol; GL: glycerol. Miscellaneous (Misc.) contains AMP (adenosine monophosphate) and LPIM (lyso-phosphoinositolmonomannoside). Source data are provided as a Source Data file.

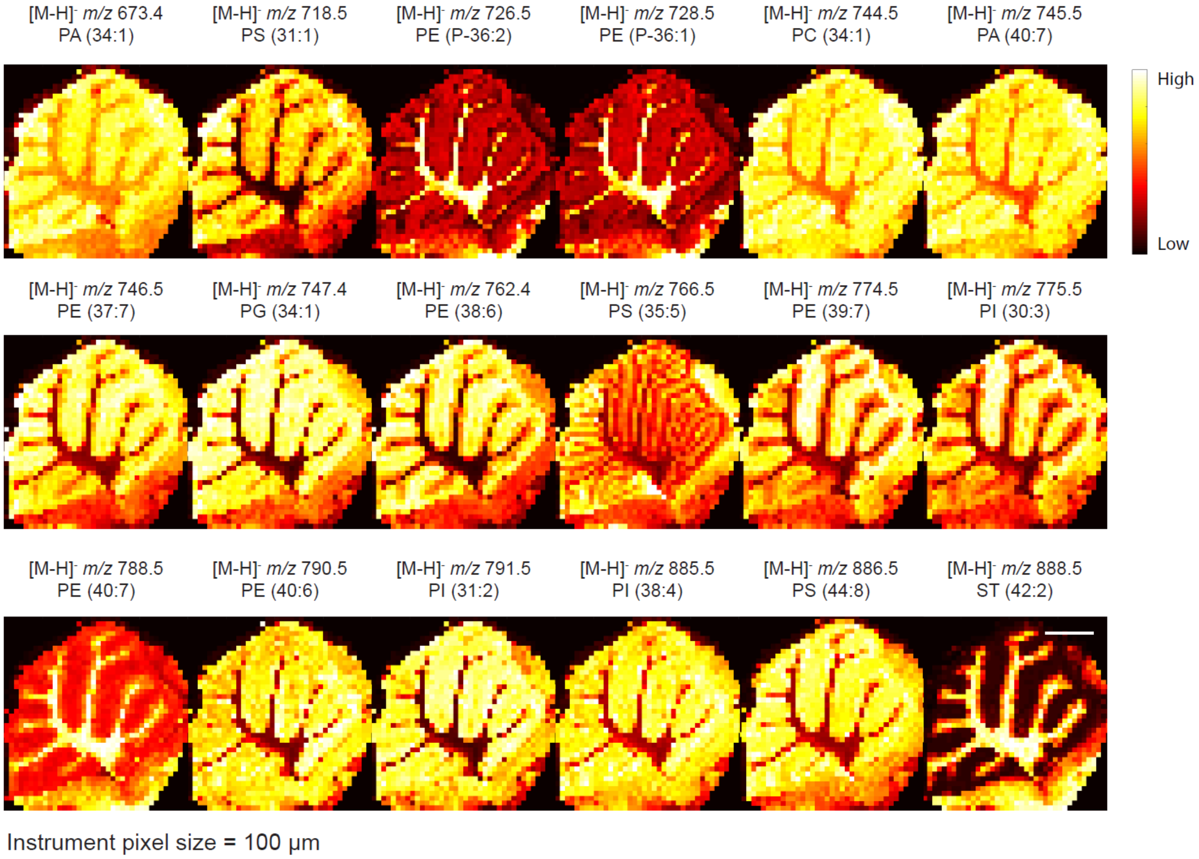

Supplementary Fig. 7: Spatial distributions of selected lipids in fresh-frozen mouse cerebellum. The sample was imaged using an AB SCIEX 4800 with an instrument pixel size of 100 μm. Scale bar: 1 mm. Here and after, unless otherwise noted, color scale bars for mass spectrometry images represent the relative intensity of the signals and *m/z* (mass-to-charge ratio) values are provided for singly charged deprotonated ions $[M-H]^-$. PA: phosphatidic acid; PS: phosphatidylserine; PE: phosphatidylethanolamine; PG: phosphatidylglycerol; PI: phosphatidylinositol; PC: phosphatidylcholine; ST: sterol.

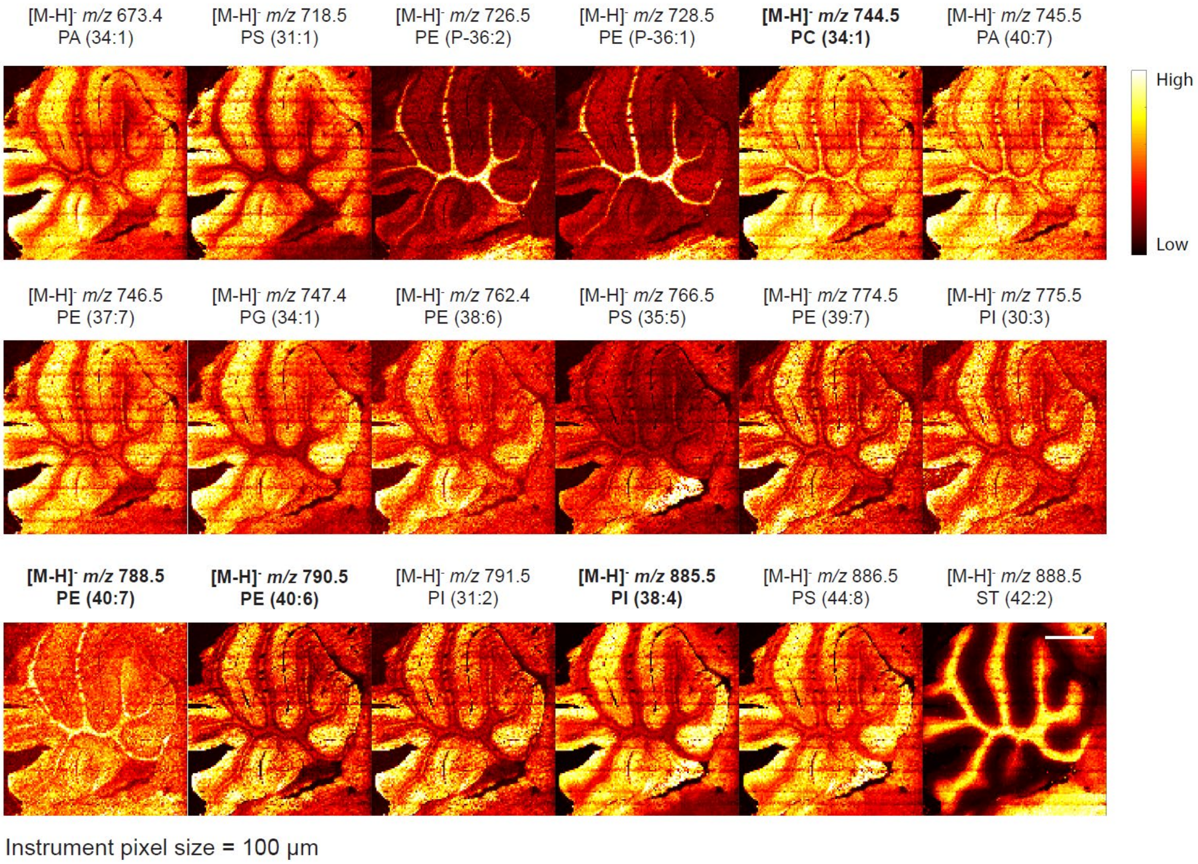

Supplementary Fig. 8: Spatial distributions of selected lipids from lipid GAMSI of PFA/GA-fixed mouse cerebellum. The GAMSI-processed sample was imaged using an AB SCIEX 4800 with an instrument pixel size of 100 µm. Scale bar: 1 mm (3 mm). Lipid names include both those assigned using the lipid database LIPID MAPS and confirmed by MS/MS (bolded). PA: phosphatidic acid; PS: phosphatidylserine; PE: phosphatidylethanolamine; PG: phosphatidylglycerol; PI: phosphatidylinositol; PC: phosphatidylcholine; ST: sterol.

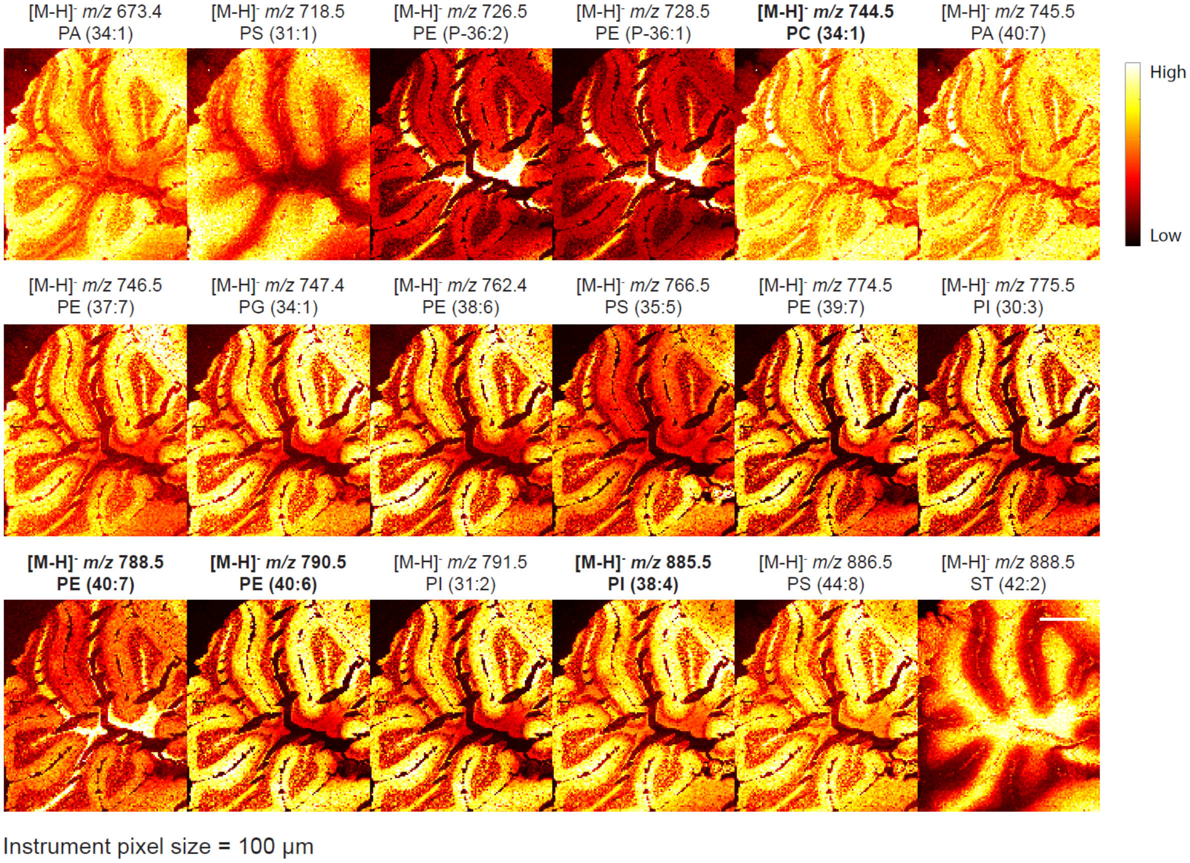

Supplementary Fig. 9: Spatial distributions of selected lipids from lipid GAMSI of PFA-fixed mouse cerebellum. The GAMSI-processed sample was imaged using an AB SCIEX 4800 with an instrument pixel size of 100 µm. Scale bar: 1 mm (4 mm). Lipid names include both those assigned using the lipid database LIPID MAPS and confirmed by MS/MS (bolded). The apparent tissue tears are from cryo-sectioning. PA: phosphatidic acid; PS: phosphatidylserine; PE: phosphatidylethanolamine; PG: phosphatidylglycerol; PI: phosphatidylinositol; PC: phosphatidylcholine; ST: sterol.

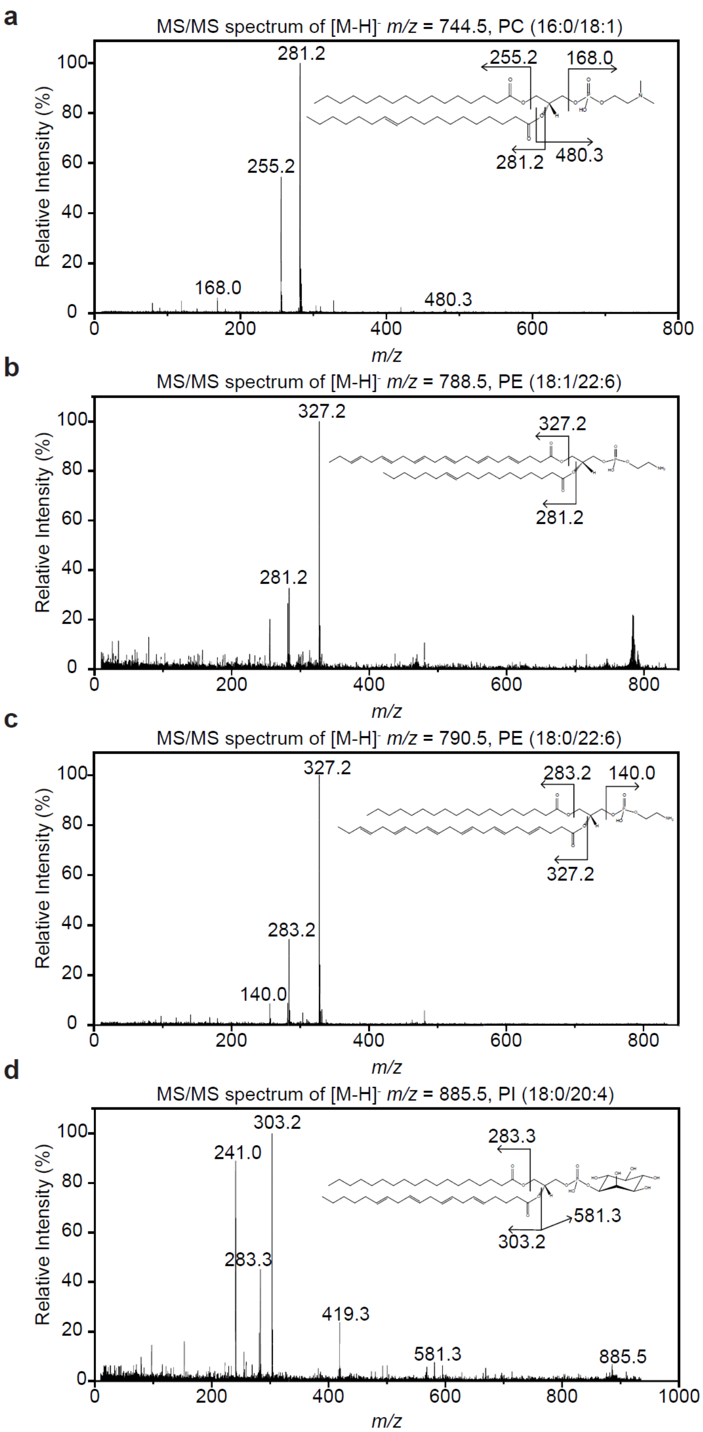

Supplementary Fig. 10: Tandem mass spectrometry (MS/MS) validation. On-tissue MS/MS spectra of representative lipid peaks at *m/z* = (**a**) 744.5, (**b**) 788.5, (**c**) 790.5, and (**d**) 885.5 from GAMSI-processed mouse cerebellum (PFA/GA-fixed) were collected using an AB SCIEX 4800 with an instrument pixel size of 100 µm. PC: phosphatidylcholine; PE: phosphatidylethanolamine; PI: phosphatidylinositol. Source data are provided as a Source Data file.

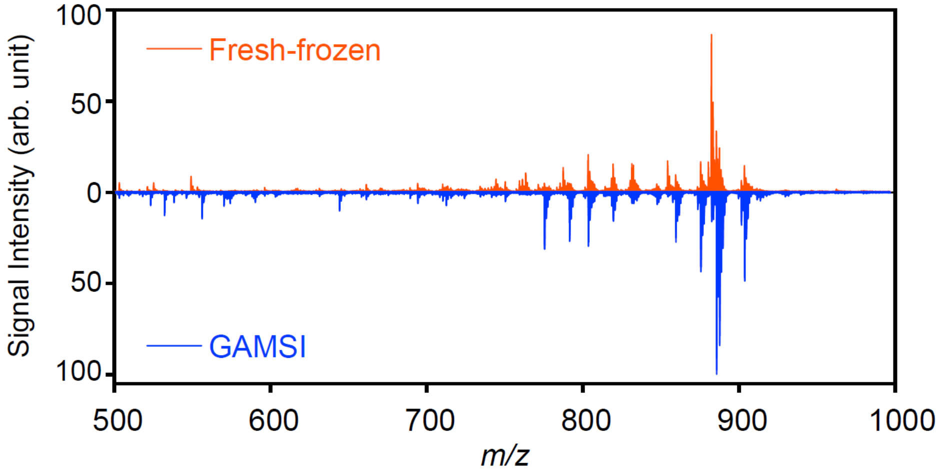

Supplementary Fig. 11: Averaged mass spectra (*m/z* = 500-1000) of fresh-frozen and GAMSI-processed (PFA-fixed) mouse cerebellum. The mass spectra were collected using a Bruker rapifleX MALDI Tissuetyper ("Bruker rapifleX") using 9AA as the default matrix for lipid imaging with an instrument pixel size of 50 µm. Source data are provided as a Source Data file.

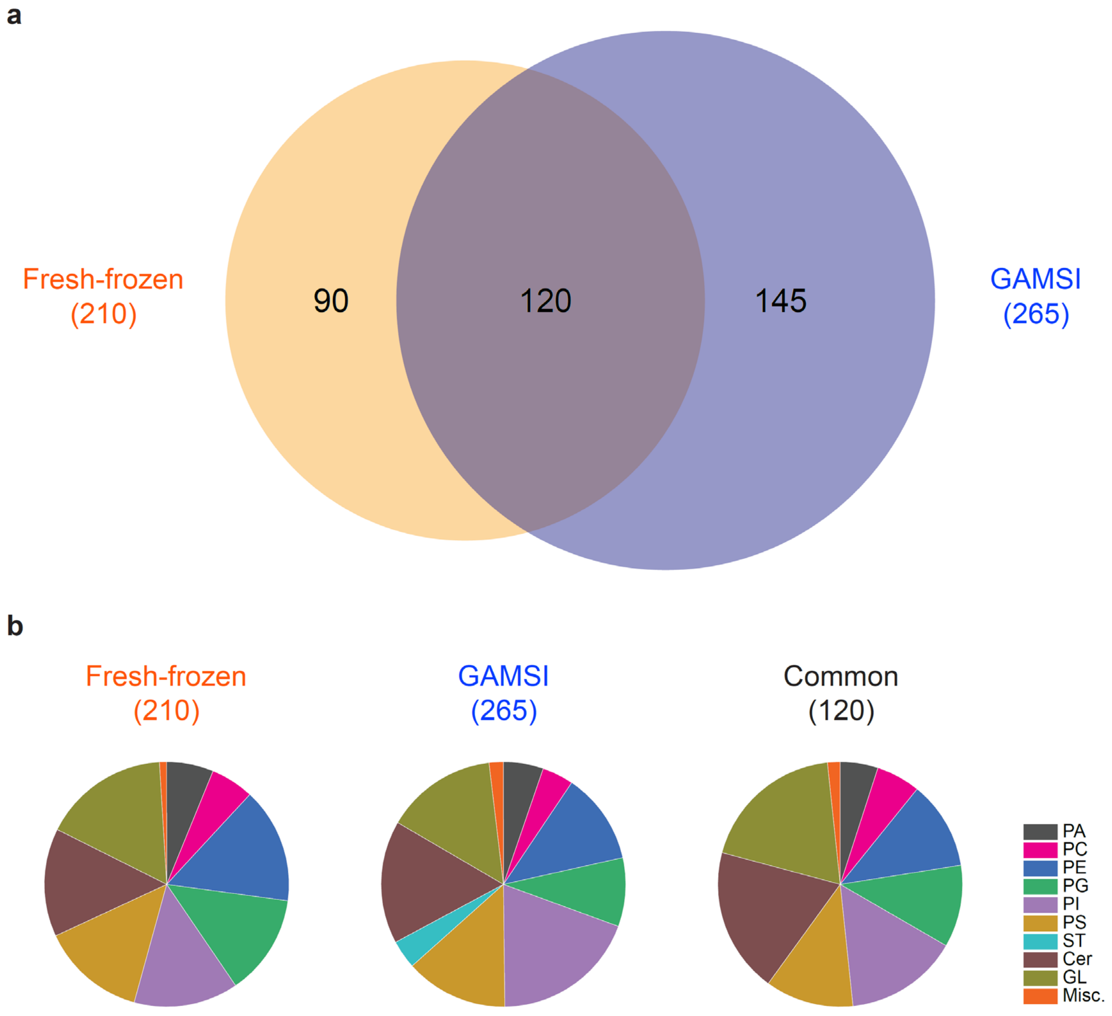

Supplementary Fig. 12: Lipid profile of fresh-frozen and GAMSI sample (Bruker rapifleX). a, Venn diagram showing common and different lipid peaks from averaged mass spectra of fresh-frozen and GAMSI-processed (PFA-fixed) mouse cerebellum using a Bruker rapifleX. All lipid assignments were made by comparing the lipid peaks to the LIPID MAPS database with an allowed mass tolerance of *m/z* = +/- 0.05. **b,** Pie chart showing the chemical composition of all the fresh-frozen peaks (left), GAMSI peaks (center), and common peaks (right). Instrument pixel size was set at 50 µm. PA: phosphatidic acid; PC: phosphatidylcholines; PE: phosphatidylethanolamine; PG: phosphatidylglycerol; PI: phosphatidylinositol; PS: phosphatidylserine; ST: sterol; GL: glycerol. Miscellaneous (Misc.) contains Glc-GP (glycosylglycerophospholipid). Source data are provided as a Source Data file.

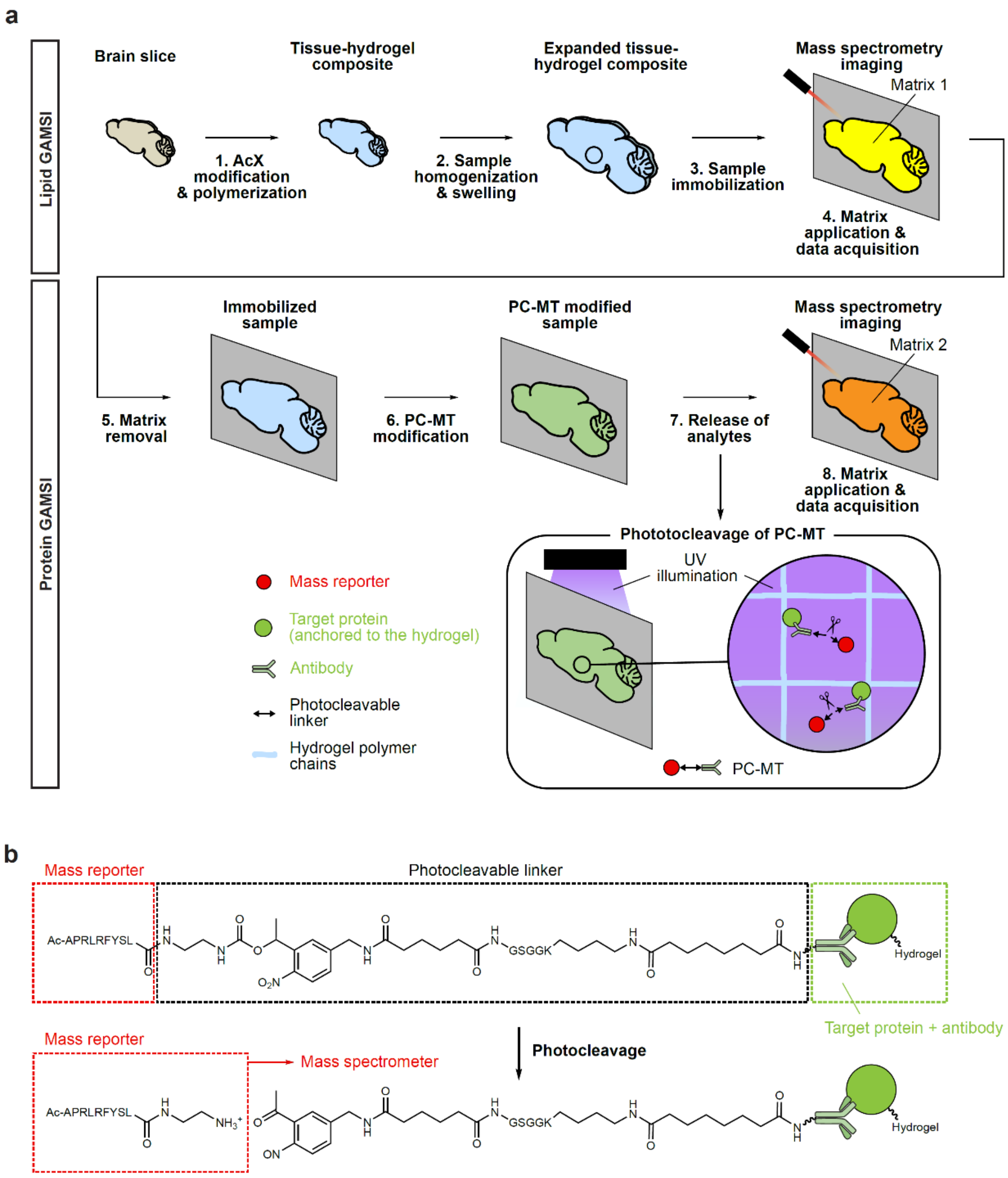

Supplementary Fig. 13: Multiplexed lipid-protein GAMSI workflow. a, Schematics showing the sequential workflow of lipid and protein GMASI. Brain slices are (1) treated with a small-molecule linker Acryloyl-X, SE (AcX) and polymerized to form a superabsorbent hydrogel composite, (2) homogenized and expanded, (3) immobilized onto a sample plate, (4) coated with a matrix (Matrix 1) and analyzed on a MALDI-TOF mass spectrometer for lipid imaging, (5) stripped of the matrix, (6) treated with antibody-conjugated photocleavable mass-tags (PC-MTs), (7) subject to photocleavage of PC-MTs to release the mass reporters (solid box), and (8) coated with a matrix (Matrix 2) and analyzed on a MALDI-TOF mass spectrometer for targeted protein imaging. The magnified schematic (solid circle) shows the release of the mass reporters from the target protein-antibody complexes under UV illumination. The target proteins are covalently anchored to the hydrogel polymer chains via AcX. **b,** Generalized design of PC-MT and its photocleavable linker.

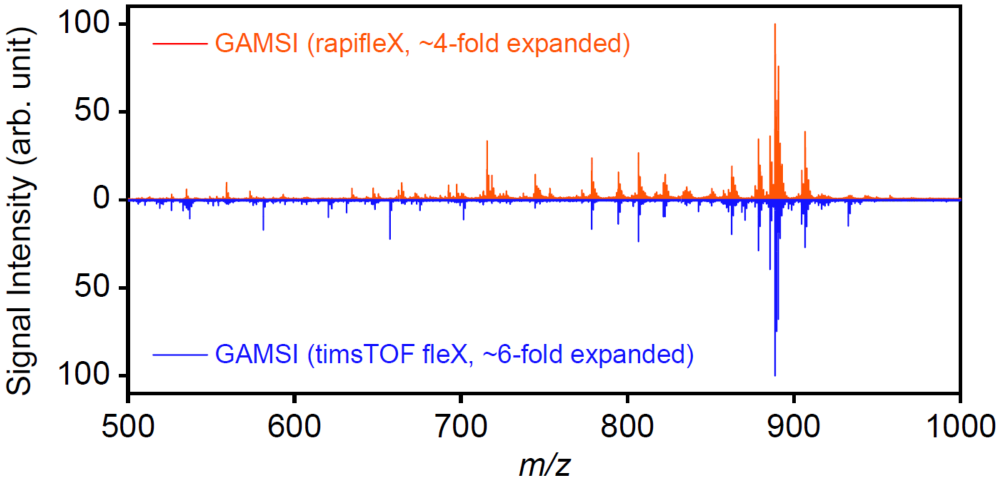

Supplementary Fig. 14: Averaged mass spectra (*m/z* = 500-1000) of ~4-fold and ~6-fold expanded GAMSI (PFA-fixed) mouse cerebellum. The mass spectra were collected using a Bruker rapifleX MALDI Tissuetyper ("Bruker rapifleX") and a Bruker timsTOF fleX MALDI-2 ("Bruker timsTOF fleX", in MALDI-1 mode) with an instrument pixel size of 50 µm and 5 µm, respectively. Source data are provided as a Source Data file.
