# Supplementary material for: Gel-assisted mass spectrometry imaging enables sub-micrometer spatial lipidomics
Source: Nat Commun. 2024 Jun 12;15:5036. doi: 10.1038/s41467-024-49384-w (PMC11169460; doi:10.1038/s41467-024-49384-w)
Supplement: Supplementary file 2 — Reporting Summary [file 41467_2024_49384_MOESM2_ESM.pdf]

nature portfolio

Corresponding author(s): Ruixuan Gao

Last updated by author(s): May 17, 2024

Reporting Summary

Nature Portfolio wishes to improve the reproducibility of the work that we publish. This form provides structure for consistency and transparency in reporting. For further information on Nature Portfolio policies, see our Editorial Policies and the Editorial Policy Checklist.

Statistics

For all statistical analyses, confirm that the following items are present in the figure legend, table legend, main text, or Methods section.

n/a	Confirmed	
[ ]	[x]	The exact sample size (n) for each experimental group/condition, given as a discrete number and unit of measurement
[ ]	[x]	A statement on whether measurements were taken from distinct samples or whether the same sample was measured repeatedly
[x]	[ ]	The statistical test(s) used AND whether they are one- or two-sided<br>*Only common tests should be described solely by name; describe more complex techniques in the Methods section.*
[x]	[ ]	A description of all covariates tested
[x]	[ ]	A description of any assumptions or corrections, such as tests of normality and adjustment for multiple comparisons
[ ]	[x]	A full description of the statistical parameters including central tendency (e.g. means) or other basic estimates (e.g. regression coefficient) AND variation (e.g. standard deviation) or associated estimates of uncertainty (e.g. confidence intervals)
[x]	[ ]	For null hypothesis testing, the test statistic (e.g. F, t, r) with confidence intervals, effect sizes, degrees of freedom and P value noted<br>*Give P values as exact values whenever suitable.*
[x]	[ ]	For Bayesian analysis, information on the choice of priors and Markov chain Monte Carlo settings
[x]	[ ]	For hierarchical and complex designs, identification of the appropriate level for tests and full reporting of outcomes
[x]	[ ]	Estimates of effect sizes (e.g. Cohen's d, Pearson's r), indicating how they were calculated

Our web collection on statistics for biologists contains articles on many of the points above.

Software and code

Policy information about availability of computer code

Data collection	Mass spectrometry data from 4800 Plus MALDI TOF/TOF Analyzer (Applied Biosystem SCIEX) were collected using 4000 Series Explorer v5.5.3 (Applied Biosystem SCIEX) and 4800 Imaging Tool v.3.2 (Applied Biosystem SCIEX).<br>Mass spectrometry data from rapifleX MALDI Tissuetyper (Bruker) were collected using flexControl 4.0 (Build 46) (Bruker).<br>Mass spectrometry data from timsTOF flex MALDI-2 (Bruker) were collected using timsControl 4.1.8 (11f8cf17) (Bruker).<br>Fluorescence microscopy data were collected using NIS Elements AR v5.30.04 (Nikon).
Data analysis	Expansion isotropy analysis was performed using "Non-Rigid Registration", a custom code implemented on MATLAB R2022a. (The custom code is available on GitHub at https://github.com/HoraceChan99/NonRigidReg.git.)<br>Fluorescence microscopy data were analyzed and visualized using ImageJ (Fiji) 1.53t.<br>Mass spectrometry imaging data were analyzed and visualized using MSiReader v1.02 or SCiLS lab v2024a (Bruker). Part of the mass spectrometry data were analyzed using MetaboScape (Bruker).<br>Mass spectra and charts were generated using OriginPro 2023 (Origin).

For manuscripts utilizing custom algorithms or software that are central to the research but not yet described in published literature, software must be made available to editors and reviewers. We strongly encourage code deposition in a community repository (e.g. GitHub). See the Nature Portfolio guidelines for submitting code & software for further information.

Data

Policy information about availability of data

All manuscripts must include a data availability statement. This statement should provide the following information, where applicable:

- Accession codes, unique identifiers, or web links for publicly available datasets
- A description of any restrictions on data availability
- For clinical datasets or third party data, please ensure that the statement adheres to our policy

The mass spectrometry data generated in this study have been deposited in the public MassIVE repository under accession code MSV000094777 [ftp://massive.ucsd.edu/v07/MSV000094777/]. Source data are provided with this paper.

Research involving human participants, their data, or biological material

Policy information about studies with human participants or human data. See also policy information about sex, gender (identity/presentation), and sexual orientation and race, ethnicity and racism.

Reporting on sex and gender	This information has not been collected.
Reporting on race, ethnicity, or other socially relevant groupings	This information has not been collected.
Population characteristics	Not applicable
Recruitment	Not applicable
Ethics oversight	Not applicable

Note that full information on the approval of the study protocol must also be provided in the manuscript.

Field-specific reporting

Please select the one below that is the best fit for your research. If you are not sure, read the appropriate sections before making your selection.

☒ Life sciences ☐ Behavioural & social sciences ☐ Ecological, evolutionary & environmental sciences

For a reference copy of the document with all sections, see nature.com/documents/nr-reporting-summary-flat.pdf

Life sciences study design

All studies must disclose on these points even when the disclosure is negative.

Sample size	The sample size used in the study was based on our past experience as well as previous studies in the field that developed similar sample expansion and imaging methods [e.g., Nat. Methods 16, 71–74 (2019), Nat. Methods 14, 593–599 (2017), Nat. Biotechnol. 34, 987–992 (2016), and Nat. Methods 13, 485–488 (2016)].
Data exclusions	This is not relevant as no data were excluded.
Replication	Unless otherwise noted, all data shown were obtained from at least three technical replicates. For representative images, unless otherwise noted, each experiment was successfully repeated at least three times under similar conditions, and the results shown are representative of the repeats.
Randomization	This is not relevant as no treatment assignments or experimental group allocations were involved in the study. All the biological samples were allocated to a single experimental group and examined as they got embedded in and expanded by hydrogels.
Blinding	This is not relevant as no treatment assignments or experimental group allocations were involved in the study. All the biological samples were allocated to a single experimental group and examined as they got embedded in and expanded by hydrogels.

Reporting for specific materials, systems and methods

We require information from authors about some types of materials, experimental systems and methods used in many studies. Here, indicate whether each material, system or method listed is relevant to your study. If you are not sure if a list item applies to your research, read the appropriate section before selecting a response.

Materials & experimental systems

n/a	Involved in the study
☐	☒ Antibodies
☒	☐ Eukaryotic cell lines
☒	☐ Palaeontology and archaeology
☐	☒ Animals and other organisms
☒	☐ Clinical data
☒	☐ Dual use research of concern
☒	☐ Plants

Methods

n/a	Involved in the study
☒	☐ ChIP-seq
☒	☐ Flow cytometry
☒	☐ MRI-based neuroimaging

Antibodies

Antibodies used	Primary antibodies:<br>Miralys ANTIBODY Probe (MBP) (AmberGen, AP1001200, 1:100)<br>Miralys ANTIBODY Probe (Synapsin I) (AmberGen, AP1001204, 1:100)<br>Rabbit anti-NF-200 (Millipore Sigma, N4142-.2ML, 1:100)<br>Rabbit anti-calbindin (Thermo Fisher, PA1-931, 1:100)<br><br>Secondary antibodies:<br>Alexa Fluor 568-conjugated goat anti-rabbit antibody (Thermo Fisher, A11011, 1:200)
Validation	Detailed analysis and species validation of the primary antibodies can be found in related publications and, for example, the following vendor websites:<br>Miralys ANTIBODY Probe (MBP) (AmberGen , AP1001200, 1:100): Highly Multiplexed Immunohistochemical MALDI-MS Imaging of Biomarkers in Tissues (https://pubs.acs.org/doi/10.1021/jasms.0c00473)<br>Miralys ANTIBODY Probe (Synapsin I) (AmberGen , AP1001204, 1:100): Highly Multiplexed Immunohistochemical MALDI-MS Imaging of Biomarkers in Tissues (https://pubs.acs.org/doi/10.1021/jasms.0c00473)<br>Rabbit anti-NF-200 (Millipore Sigma, N4142-.2ML, 1:100): https://www.sigmaaldrich.com/US/en/product/sigma/n4142<br>Rabbit anti-calbindin (Thermo Fisher, PA1-931, 1:100): https://www.thermofisher.com/antibody/product/Calbindin-D28K-Antibody-Polyclonal/PA1-931

Animals and other research organisms

Policy information about studies involving animals; ARRIVE guidelines recommended for reporting animal research, and Sex and Gender in Research

Laboratory animals	Mice (BALB/cNctr-Npc1m1N/J, Jackson Laboratory), 7 weeks old, both male and female.
Wild animals	None.
Reporting on sex	Both male and female. Sex was not considered due to the proof-of-concept nature of our study.
Field-collected samples	None.
Ethics oversight	The University of Illinois Chicago Animal Care Committee.<br>All animals were housed in appropriate cages with 12-h dark and 12-h light cycle, ambient temperature and humidity.

Note that full information on the approval of the study protocol must also be provided in the manuscript.
